# ID 212 - Análise das Contribuições em Consultas Públicas sobre Temas Avaliados pela Conitec: participação social no processo de incorporação de tecnologias em saúde

**DOI:** 10.5327/2237-9622.2025.v34s1.212

**Published:** 2025-11-25

**Authors:** Karina de Castro Zocrato, Daniel Pitchon dos Reis, Marcus Carvalho Borin, Carina Rejane Martins, Geraldo José Coelho Ribeiro, Júlia Teixeira Tupinambás, Lélia Maria de Almeida Carvalho, Marcela Pinto de Freitas, Maria da Glória Cruvinel Horta, Mariana Michel Barbosa, Mariza Cristina Torres Talim, Sergio Adriano Loureiro Bersan, Silvana Marcia Bruschi Kelles

**Keywords:** consultas públicas, Conitec, participação social, incorporação de tecnologias, saúde coletiva

## Abstract

**Introdução:**

As consultas públicas são ferramentas essenciais para garantir transparência e engajamento da sociedade no processo de tomada de decisões no Sistema Único de Saúde (SUS). A Comissão Nacional de Incorporação de Tecnologias no SUS (Conitec) utiliza consultas públicas para coletar opiniões, críticas e informações sobre suas recomendações para a incorporação de medicamentos ou outras tecnologias sanitárias. Nos últimos anos, as consultas públicas têm sido fundamentais para promover a participação social na definição das tecnologias incorporadas ao SUS. Entretanto, a análise da tendência de participação em consultas públicas é essencial para avaliar o engajamento da sociedade e identificar possíveis desafios no processo de participação. Este estudo visa analisar a tendência temporal de contribuições para consultas públicas da Conitec desde 2018, buscando identificar tendências.

**Método:**

Os dados foram coletados a partir do painel de acompanhamento de tecnologias em saúde submetidas à Conitec, acessado em 30 de agosto de 2024. Para análise, foram incluídas as contribuições a partir de 2018, quando o número total de participações superou 20 mil por ano. Os dados de 2024 foram considerados até a data de análise. Foi calculada a média de contribuições por consulta pública. com mais de 5 mil contribuições foram avaliados individualmente, mas não entraram no cálculo da média.

**Resultados:**

Entre 2018 e 2024, foram registradas aproximadamente 380 mil contribuições para as consultas públicas da Conitec. Observou-se uma queda significativa no número médio de contribuições a partir de 2021, com a média de contribuições por consulta pública caindo de 698,7 em 2021 para 337,6 em 2024. As duas consultas sobre o medicamento nusinersena foram as que mais receberam contribuições, com 41 mil em 2019 e 36 mil em 2020, respectivamente. Mesmo com a exclusão dos do cálculo da média, a tendência de redução nas contribuições se manteve.

**Conclusão:**

Os resultados indicam uma diminuição na participação da sociedade nas consultas públicas da Conitec desde 2021. Esse declínio pode refletir uma série de fatores, como mudanças no engajamento social, o impacto da pandemia de covid-19, o tipo de tecnologias em discussão, e alteração na forma de acesso para manifestação no portal da Conitec. Além disso, é importante considerar que, em algumas consultas, o engajamento pode ser influenciado por grupos com interesses específicos, cujas contribuições são mobilizadas por campanhas que atendam a esses interesses. Esses achados reforçam a necessidade de revisar as estratégias de engajamento da população às consultas da Conitec, assegurando que a sociedade em geral continue a ter voz ativa no processo de tomada de decisão sobre a incorporação de tecnologias no SUS.

